# Evolution of the Multi-Domain Structures of Virulence Genes in the Human Malaria Parasite, *Plasmodium falciparum*

**DOI:** 10.1371/journal.pcbi.1002451

**Published:** 2012-04-12

**Authors:** Caroline O. Buckee, Mario Recker

**Affiliations:** 1 Center for Communicable Disease Dynamics, Department of Epidemiology, Harvard School of Public Health, Boston, Massachusetts, United States of America; 2 Department of Zoology, University of Oxford, Oxford, United Kingdom; Emory University, United States of America

## Abstract

The *var* gene family of *Plasmodium falciparum* encodes the immunodominant variant surface antigens PfEMP1. These highly polymorphic proteins are important virulence factors that mediate cytoadhesion to a variety of host tissues, causing sequestration of parasitized red blood cells in vital organs, including the brain or placenta. Acquisition of variant-specific antibodies correlates with protection against severe malarial infections; however, understanding the relationship between gene expression and infection outcome is complicated by the modular genetic architectures of *var* genes that encode varying numbers of antigenic domains with differential binding specificities. By analyzing the domain architectures of fully sequenced *var* gene repertoires we reveal a significant, non-random association between the number of domains comprising a *var* gene and their sequence conservation. As such, *var* genes can be grouped into those that are short and diverse and genes that are long and conserved, suggesting gene length as an important characteristic in the classification of *var* genes. We then use an evolutionary framework to demonstrate how the same evolutionary forces acting on the level of an individual gene may have also shaped the parasite's gene repertoire. The observed associations between sequence conservation, gene architecture and repertoire structure can thus be explained by a trade-off between optimizing within-host fitness and minimizing between-host immune selection pressure. Our results demonstrate how simple evolutionary mechanisms can explain *var* gene structuring on multiple levels and have important implications for understanding the multifaceted epidemiology of *P. falciparum* malaria.

## Author Summary

*Plasmodium falciparum*, the most severe of the human malarias, contains within its genome a family of ∼60 *var* genes which play an important role in disease pathology and maintenance of chronic infections. *Var* genes have a modular genetic architecture and encode varying numbers of binding domains with specific preferences to a range of host tissues. Given the availability of host receptors for binding and the immunogenic properties of each domain it is not clear why genes encode multiple domains at once and how these are structured within each parasite's antigenic repertoire. Here we investigate the domain architecture of these important virulence genes and highlight an evolutionary trade-off between maintaining within-host fitness and optimizing between-host transmission success as an important driver in structuring *var* genes and *var* gene repertoires alike.

## Introduction

The malaria parasite *Plasmodium falciparum* continues to be a major public health problem globally [1], [2], [3]. In malaria-endemic regions, repeated infections by genetically diverse parasites continue into adulthood, and clinical protection appears to rely on the gradual acquisition of variant-specific immunity to polymorphic antigens [4], [5], [6]. The major target for this protective immunity is thought to be PfEMP1 (*Plasmodium falciparum* erythrocyte membrane protein 1), expressed on the surface of infected red blood cells (iRBC) and encoded by the *var* multi-gene family. PfEMP1 mediates adhesion to a variety of host receptors on the endothelium as well as to uninfected red blood cells, leading to the sequestration of parasitized cells in peripheral blood vessels and organs [7], [8], [9], [10], [11]. This prevents the parasite from being cleared by the spleen [12], [13], however it is also associated with a range of severe disease pathologies [14], [15], [16]. Switches in expression between the parasite's repertoire of ∼60 *var* genes during within-host antigenic variation, a process which facilitates immune evasion, may therefore alter its binding phenotype and consequently the pathology of infection [17], [18], [19]. The relationship between mechanisms of acquired immunity, disease outcomes, and the evolution and structure of these antigens are still poorly understood, despite the critical role these interactions play in shaping the epidemiology of malaria.

**Figure 1:**
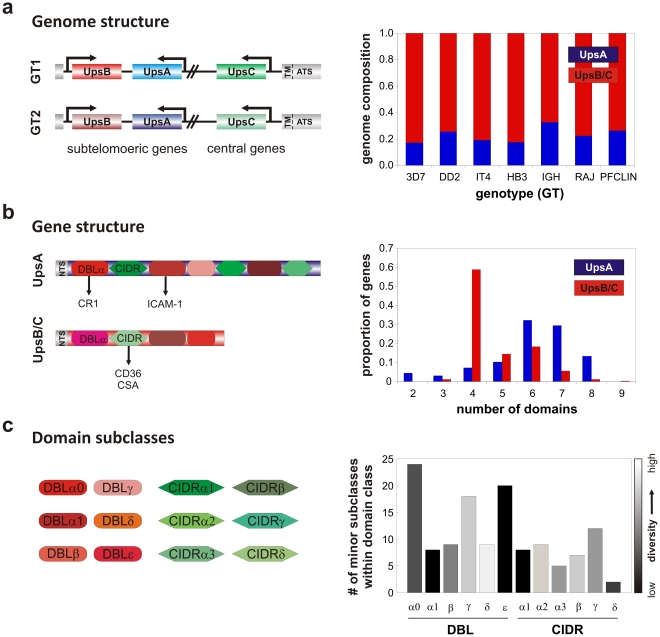
Schematic illustrating the hierarchical organization of *var* domains, genes, and genome repertoires. ***a***) *Var* genes can be classified by their chromosomal location and upstream promoter (Ups) type into subtelomeric groups A and B and the central group C. The proportion of genes belonging to these groups in a parasites genome is well conserved across different isolates, with the majority of genes belonging to groups B or C (red bars). ***b***) Individual genes are comprised of a varying number of DBL and CIDR domains which mediate binding to different host receptors in a group-dependent fashion. Both groups (UpsA and non-UpsA) show widely distributed domain architectures; however, the majority of long genes, i.e. those with >6 domains, belong to group A (blue bars) whereas most B/C genes (red bars) consist of 4 domains. ***c***) Binding domains can further be divided into various sub-classes and there is extensive variation in terms of frequency and sequence diversity on a population level.

One of the most striking characteristics of PfEMP1 is the large, variable domain structure of the extracellular portion of the protein [20]. *Var* genes are characterised by variable numbers of diverse Duffy Binding Like (DBL) domains and Cysteine-rich InterDomain Regions (CIDRs), which can be further divided into a number of subclasses, arranged in different combinations (see schematic in Figure 1). The extremely high diversity of *var* gene sequences, generated by means of recombination within and between genomes [21], [22], has made it difficult to analyse the structure of individual parasite *var* repertoires and the parasite population as a whole. Due to the difficulty in designing suitable primers, v*ar* genes have traditionally been grouped according to upstream promoter regions (Ups) A, B, and C, roughly corresponding to the direction of transcription and the chromosomal location [23], as well as short sequence fragments within the relatively conserved N-terminal DBLα domain [24]. Recently, however, whole *var* repertoires from seven genomes have been sequenced, revealing a remarkable variety of PfEMP1 structures while confirming that repertoires from geographically diverse parasites are nonetheless structured in a similar way [25]. A classic example of this shared structure is the preservation of one or two copies of *var2csa*, which encodes a PfEMP1 variant that specifically mediates binding to placental Chondroitin Sulfate A [26], [27] and has been found in every parasite genome studied to date. The remaining *var* gene repertoire is composed of a mixture of long (primarily Ups group A) and short (mostly Ups group B and C) *var* genes in approximately similar proportions, as shown in Figure 1a and b.

The specialization of *var2csa* for a particular ecological niche has led to the idea that the composition of each repertoire with respect to the other groups of *var* genes may reflect similar specialization for different host niches and binding phenotypes. For example, UpsB and UpsC genes have been shown to bind ICAM1 and CD36, mediated by DBLβC2 [28] and CIDRα domains [29], respectively, whereas in field isolates UpsA genes (and some ICAM1 binders) are involved in the binding to uninfected erythrocytes [30] via the DBL1α domain to Complement Receptor-1 (CR1) [31], a process known as rosetting and implicated in severe malarial disease among young children [32]. This suggests that specific genes are adapted to particular host niches, with certain domains within those genes being responsible for the specific binding properties. However, the specialization of individual domains for particular host receptors, rather than entire genes, raises important questions about the structuring and evolution of variable domain architectures. Specifically, how does the structure of domains within *var* genes relate to *var* genome repertoires and parasite fitness, and what evolutionary processes can account for this structuring?

There are two important but largely unexplored aspects of *var* genes which make them unusual in the context of host-pathogen systems. First, most theoretical models of pathogen population structure consider (immune) selection operating on a between-host level through cross-immunity that depends on a degree of antigenic similarity or overlap between pathogenic variants [33], [34], [35], [36]. Under these assumptions pathogens will evolve to minimise immunological interference, favouring variants with fewer antigenic targets. The large modular domain architecture of *var* genes is therefore difficult to explain, considering that each domain presents an additional target to the immune system and that PfEMP1 plays a key role in both immune evasion and naturally acquired immunity. Although the exact mechanisms of immune protection to malaria are still unclear, antibodies to PfEMP1 are thought to generate the primary protective responses to blood stage infections, and have been shown to inhibit cytoadhesion of infected red blood cells in a domain specific manner [28], [29], [37], [38], [39], [40], [41], [42]. We hypothesize that this type of immunity would not block infection or preventing transmission entirely but contribute to parasite removal by preventing sequestration of parasitized red cells. Under these circumstances, additional binding domains could provide some residual cytoadherent properties even when pre-existing or cross-reactive immune responses are present, offering a within-host fitness advantage. Thus, both between- and within-host selection pressures (optimal binding vs. antigenic diversity) may be operating on *var* genes in conflicting ways.

The second important consideration in understanding the structure of *var* genes and *var* gene repertoires is phenotypic change through the process of antigenic variation. It has long been recognized that the cytoadhesion phenotype of a particular malaria parasite exhibits “plasticity” [43], which is likely to depend on a combination of parasite and host genetics and the presence of antibodies, and may contribute to the observed age- and exposure dependent pathologies of malaria. Although the possibility of displaying different phenotypes in different hosts will have an important impact on the population structure of *var* gene repertoires, most theoretical approaches have so far ignored phenotypic flexibility. For example, within-host models usually concentrate on antigenic change during infection [44], [45], [46], [47], whereas some strain-theoretical, between-host models have assumed *P. falciparum* population to consist of discrete phenotypes in order to explain how the wide range of associations with disease could be contained within one pathogen population [48], [49], [50], [51]. What remains unclear, however, is how repertoires of *var* genes with different phenotypic characteristics have evolved, given that genes, and thus phenotypes, can be expressed independently.

Here we explore these two aspects of *var* gene evolution explicitly through a combination of data analysis and the development of evolutionary frameworks. First, we analyzed the *var* gene repertoires of seven fully sequenced *P. falciparum* parasite genomes recently published by Rask *et al.* [25]. Our analysis reveals that genetic architectures and domain diversities vary widely within and between different *var* gene groups but exhibit a surprising, non-random association between sequence diversity and gene length that extends beyond promoter-associated groups. We then develop an evolutionary framework to demonstrate how contrasting within- and between-host evolutionary mechanisms that operate differently at the phenotypic and genotypic level could have shaped individual domain structures as well as genomic repertoires of these important virulence genes.

## Results

### *Var* domain sequence diversity

As a result of frequent recombination and gene conversion events, *var* genes show a high degree of mosaicism [22], [52]. Previous analyses have shown that the DBLα domains of UpsA genes share more sequence mosaics with one another than the UpsB *var* genes, suggesting that this group of genes is evolutionary isolated and more conserved than other groups [21]. Here we analyzed entire genes, comparing the sequence diversity of all domains within and between *var* genes of seven fully sequenced repertoires. We used the sequence conservation (percent identity) reported in [25] as a proxy for the population-level diversity of each subclass of DBL (α, β, γ, δ and ε) and CIDR (α, β, γ, δ) domains, since conservation varied considerably within broader classes. For clarification, we refer to diversity as a measure of sequence variation whereas frequency is solely used in reference to the number or prevalence of domains within a gene or population.

**Figure 2:**
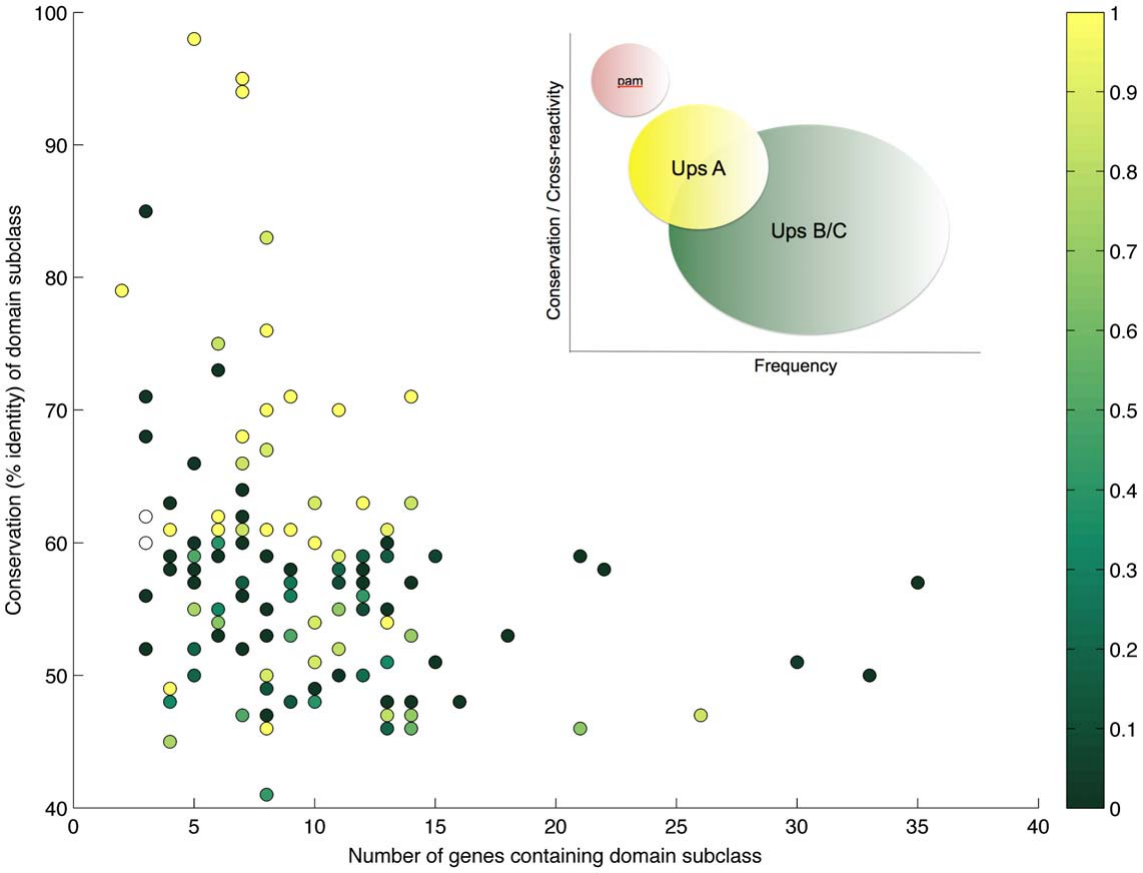
Relationship between sequence conservation, Ups group, and frequency in the data set for each domain subclass. Each dot represents a domain subclass, as classified by Rask *et al.* [25], with the colour of the dot representing the association of that subclass with Ups group (yellow dots are only found in UpsA *var* genes, dark green dots are only found in UpsB or C *var* genes, and intermediate colours are found in all Ups groups in various proportions). **Inset: Hypothetical population distribution of** ***var*** **genes.** It has been suggested that UpsA *var* genes are more conserved because they are optimized for functionality in naïve individuals, whereas UpsB and C *var* genes are more prevalent (both in the population and within *var* gene repertoires) but more diverse and can sustain infection in individuals with previous exposure [21]. This would lead to a hypothetical distribution of genes that follows a similar pattern to the domain subclasses observed here. Note that pregnancy associated malaria *var* genes (denoted ‘pam’ in the figure) represent an extreme case of this pattern.

To investigate a possible association between the frequency of domain subclasses and their population-level diversity we plotted the number of genes that contain a particular subclass against its sequence conservation for all domain subclasses found in the data set. As shown in Figure 2 we found a strong negative relationship (*R* ^2^ = −0.4, *P*<0.0001) between a domain's frequency and its sequence conservation. Note, this negative relationship is independent of Ups type and still holds when considering subclasses associated with UpsA and UpsB/C genes separately (see Supplementary Figure S1). In agreement with previous analyses based on the DBLα region only [21] we found that the most conserved domain subclasses were primarily in UpsA genes and the most diverse subclasses were found in UpsB genes (with yellow circles in Figure 2 representing domains found only in UpsA genes, dark green representing only UpsB or C, and the spectrum in between representing the proportion found within each group). Importantly, however, there is considerable overlap and both groups share many domain subclasses with a wide range of sequence diversities (see Supplementary Materials Figure S1). Bull *et al.* [53], [54] have previously reported the existence of so called ‘common’ and ‘rare’ PfEMP1 variants, based on their serological recognition profiles, and these sets of variants are thought to correspond broadly to UpsA and UpsB or C *var* genes, respectively. This is consistent with our finding that UpsA genes, or rather domains associated with UpsA genes, are on average more conserved than domains belonging to other Ups groups. Based on these results we hypothesize a relationship between the frequency of *var* gene groups and their average degree of diversity in the population which is illustrated in the inset in Figure 2.

**Figure 3:**
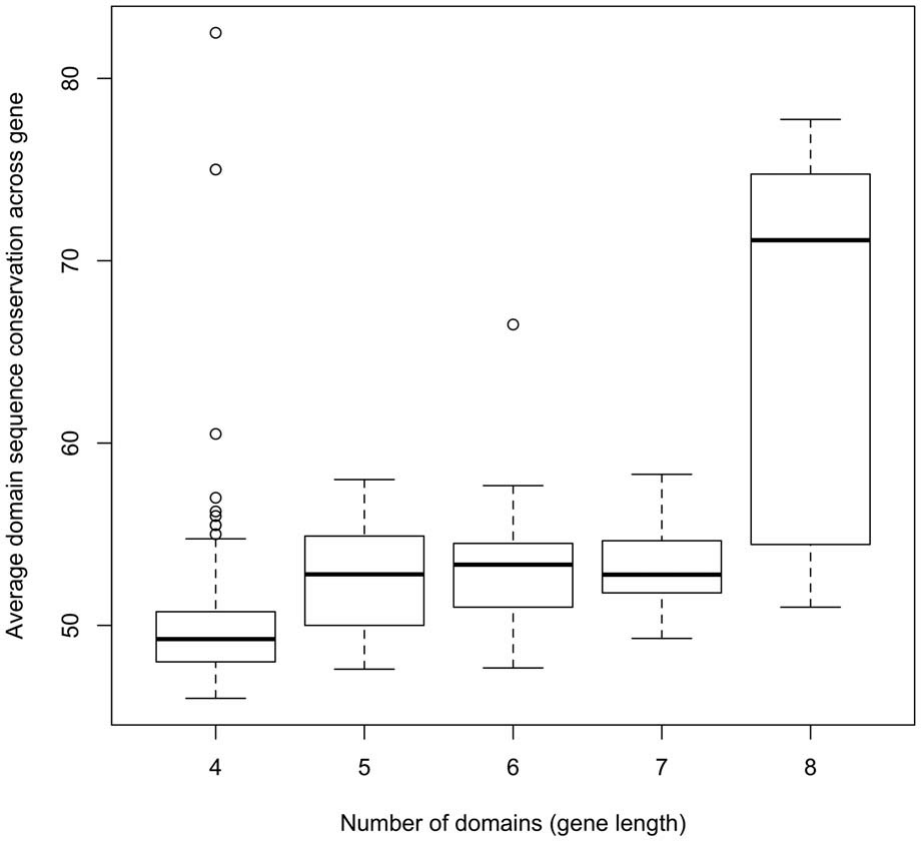
Average sequence conservation of domains across genes of different domain-length. Boxplots show the median, 1st and 3^rd^ quantiles (box limits), and upper and lower bounds for the 95% confidence intervals (whiskers, with dots as outliers) for average conservation (% identity) of domains occurring across the same *var* genes of different lengths in the Rask *et al.* [25] data.

The fact that UpsA genes appear to be recognized commonly, but also occur less frequently in the genome and on a population level, has led to the notion that UpsA genes are preferentially expressed in young, non-immune individuals, possibly due to an *in vivo* growth advantage over other Ups groups. Here we hypothesized that this type of growth advantage might be mediated by enhanced parasite binding. This idea is supported by the fact that group A genes are generally composed of more, and more conserved binding domains than genes belonging to groups B or C (Figure 1b), which in turn suggests that the reduced diversity of group A genes may be due to a functional, i.e. binding, constraint. We therefore analyzed how domains are grouped into genes, and how domain diversity is related to *var* gene length (in terms of number of domains). Figure 3 shows that longer genes contain more conserved domains on average, independent of Ups type. That is, the average sequence conservation of domain subclasses within a gene increased with the number of domains for all Ups groups (R = 0.49, *P*<1×10^−16^), suggesting that gene length might be another useful characterization of *var* gene groups besides chromosomal location or upstream promoter type. Importantly, this relationship also held when considering Ups B's alone (R = 0.42, p<1×10^−12^). Furthermore, we found that the population-level diversity of all domains found within the same gene was also strongly correlated in a pair-wise fashion, regardless of the relative positions of those domains (see Table S1 in the Supplementary Materials).

To further demonstrate these non-random associations between domain diversities across the genes, we compared the observed arrangement of domains within *var* genes to simulated data generated by randomizing the location of particular domains across genes but keeping the distribution of gene lengths constant (see Figure S2 in Supplementary Materials). As expected, the correlation between gene length and sequence conservation was not found in the randomized data sets. More importantly, though, in the randomized data the overall *var* population diversity was decreased, whereas the sequence diversity of domains within an individual *var* gene were no longer similar.

Together, these results suggest that *var* genes are organized such that domains of similar population-level diversities are grouped into the same gene. Overall, genes appear to be largely either long and conserved or short and diverse, independent of Ups type. This non-random structuring suggests an evolutionary trade-off which has shaped the *var* genes on a gene level into those we expect to be commonly recognized - due to a high number of conserved domains that facilitate optimal cytoadhesion - and those that may be rarely recognized because they contain fewer, more diverse domains and thus appear to be optimized for immune evasion. Crucially, this trade-off is possible at the gene (rather than the genome) level because *var* genes are expressed independently of each other; for example, a parasite may theoretically express its UpsA genes in one host and its UpsB or C genes in another. This phenotypic plasticity means that a single genotype (or gene repertoire) can simultaneously benefit from a range of different evolutionary strategies.

The observed domain associations within *var* genes and the structure of *var* gene repertoires open up a number of important questions: firstly, how are long *var* genes maintained in the population given that each additional domain increases the number of immune targets exposed? And secondly, what evolutionary processes can explain the non-random domain structures and genome repertoires described above? Specifically, how do genomes evolve to include *var* genes of contrasting diversities whereas *var* genes evolve to include domain subclasses with similar levels of diversity? We developed a series of related theoretical frameworks to address these questions. In the models described below we make an important distinction between pathogen phenotypes, characterized by particular *var* genes composed of one or more binding domains, and pathogen repertoires or genotypes, which may contain multiple *var* genes and possible phenotypes.

### The evolution of antigens with multiple binding domains

We first examine the conditions leading to the evolution of multiple antigenic domains. We developed a simple mathematical model where pathogen phenotypes are defined by antigens consisting of either one or two binding domains, with each domain capable of eliciting a protective, allele-specific immune response (see Methods). We assumed that the primary function of binding is to reduce parasite clearance and therefore to increase the duration of infection. We varied the type of allele-specific immunity in the model between two hypothetical extremes: i) the traditional assumption in which antibodies to one pathogen phenotype prevent infection or transmission by phenotypes sharing domains (via a high degree of cross-immunity, δ), and ii) the cytoadhesion-blocking assumption in which antibodies to a particular phenotype inhibit binding in a domain-specific manner and thus reduce transmission of antigenically related phenotypes by shortening their duration of infection (see Methods).

**Figure 4:**
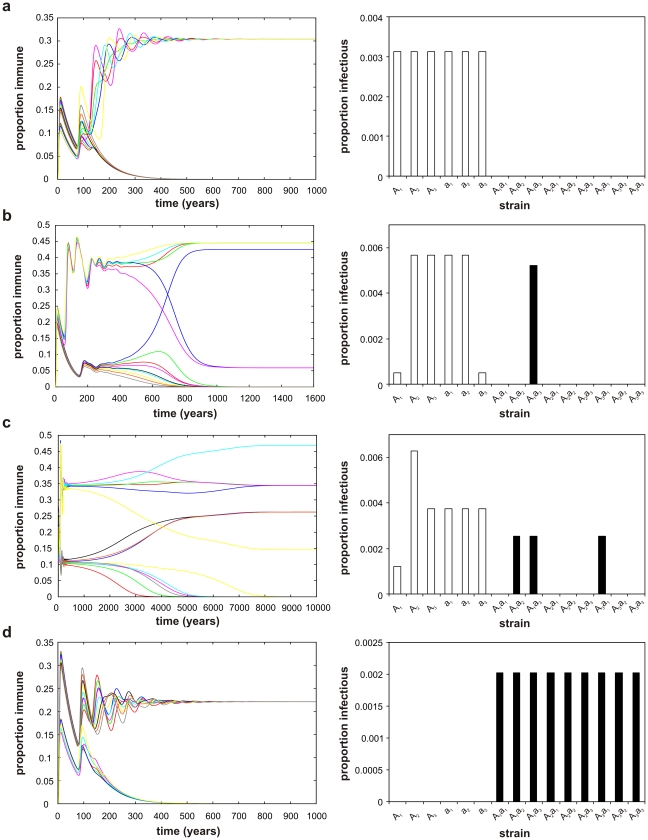
Impact of different mechanisms of immunity on multi-domain structure. Changing the assumption about the nature of immunity (broadly cross-reactive vs. domain-specific) will select for different combinations of single and multi-domain phenotypes, where each phenotype is defined by one or two binding domains, *A* and *a*, each with three alleles (*A*_1_,…, *A_3_*, *a_1_*, …, *a_3_*). *Left panel*: the dynamics of the proportion of individuals immune to specific phenotypes over time (each variant is represented by a different colour); *right panel*: the equilibrium prevalence distribution of all phenotypes (open bars: single domain types, solid bars: multi-domain types). ***a***) Traditional cross-immunity only, where immunity to one phenotype confers partial or full immunity to any phenotype sharing alleles, will lead to competitive exclusion of multi-domain types and the persistence of single domain types. ***b***, ***c***) Assuming both types of immunity acting in synergy leads to dominance of a mixture of single and multi-domain phenotypes with minimal overlap between their antigenic alleles. ***d***) Adhesion-blocking immunity only, where immunity blocks binding in a domain-specific and allele-specific manner, will lead to the complete exclusion of single domain phenotypes and the persistence of multi-domain phenotypes. Parameter values: *β* = 2, *σ_i_* = 1, *μ* = 1/70, ***a***) *δ_1_* = 2, *δ_2_* = 2.2, ***b***) *δ_1_* = 1.2, *δ_2_* = 2, ***c***) *δ_1_* = 1.1, *δ_2_* = 1.7, ***d***) *δ_1_* = 1.2, *δ_2_* = 1.5.

In our model each domain, denoted *A* and *a*, mediates binding to a different host receptor and comes in a number of antigenically related alleles, *A_i_* and *a_i_*. Assuming three alleles for each domain, for example, gives rise to a total of 15 possible pathogen phenotypes: 6 single-domain types (*A_1_*, *A_2_*, …, *a_3_*) plus 9 multi-domain types (*A_1_a_1_*, …, *A_3_a_3_*). Figure 4a shows how under the traditional formulation of cross-immunity, where the price of sharing antigenic alleles is high due to competition for susceptible hosts, phenotypes with more than one binding domain are significantly disadvantaged and outcompeted by those expressing a single-domain. In contrast, assuming that immunity predominantly prevents binding in a domain-specific manner (Figure 4d), such that antibodies from previous exposure to *A_1_* do not protect against infection with *A_1_a_1_*, for example, phenotypes with more than one binding domain can retain some cytoadhesive properties and thus gain a competitive advantage over single domain phenotypes. This leads to the dominance of multi-domain phenotypes in the population and competitive exclusion of types with only a single binding domain. If we assume that both categories of immunity play a (synergistic) role, which is probably the most realistic scenario for PfEMP1, both single and multi-domain antigens can coexist. Here, the balance between them will determine the precise population structure, but in all cases the system evolves towards a mixed structure of both single and multi-domain phenotypes with minimal allelic overlap between their antigenic loci (Figure 4b,c).

Note that if we assume that antigen repertoires (i.e. genotypes) should reflect the population-level structures modelled here, our results suggest that pathogen genomes should evolve to comprise genes with varying numbers of binding domains, and could therefore offer a very simple explanation of the evolution of the wide distribution of gene lengths observed across *var* genes from global isolates (Figure 2). Again, this argument rests on the fact that individual *var* genes within a repertoire may be expressed as independent phenotypes.

### The evolution of domain associations and gene repertoire structures

We next modified our model framework into a stochastic, individual-based model to accommodate higher degrees of antigenic and phenotypic diversity (see Methods). This model also allowed us to move easily between two biological levels of selections (at the gene and genome level) and to ask whether similar simple assumptions about immunity can explain the observed structures and association among (*i*) domains within genes (phenotype) and (*ii*) genes within genomes (genotype).

In this model, each pathogen was comprised of two antigenic variants. We used the same assumptions about immunological protection in both the phenotype and genotype models, but we varied the way in which the two antigenic variants (representing domains in the first model and genes in the second) jointly determined pathogen fitness and how it is affected by host immunity. For the two-domain or phenotype model, we assumed that the parasite clearance rate is determined by a synergistic interaction of the constituent binding domains, since the domains are always expressed together. In contrast, in the two-gene or genotype model the clearance rate was defined by the gene with the highest binding affinity to which there were no antibodies, thus representing the idea of a flexible phenotype. We considered different domains, or genes, to have different levels of sequence diversities which determined both binding affinity and cross-reactivity (i.e. antigenic relatedness). That is, we assumed that more conserved domains/genes had a within-host advantage, due to improved cytoadhesion and therefore longer periods of infections, but were disadvantaged on a population level due to higher immune selection pressure. Fitness was therefore governed by the combination of binding domains or genes and balanced by the trade-off between the within-host and within-population levels of selection.

### Evolution of domain associations

**Figure 5:**
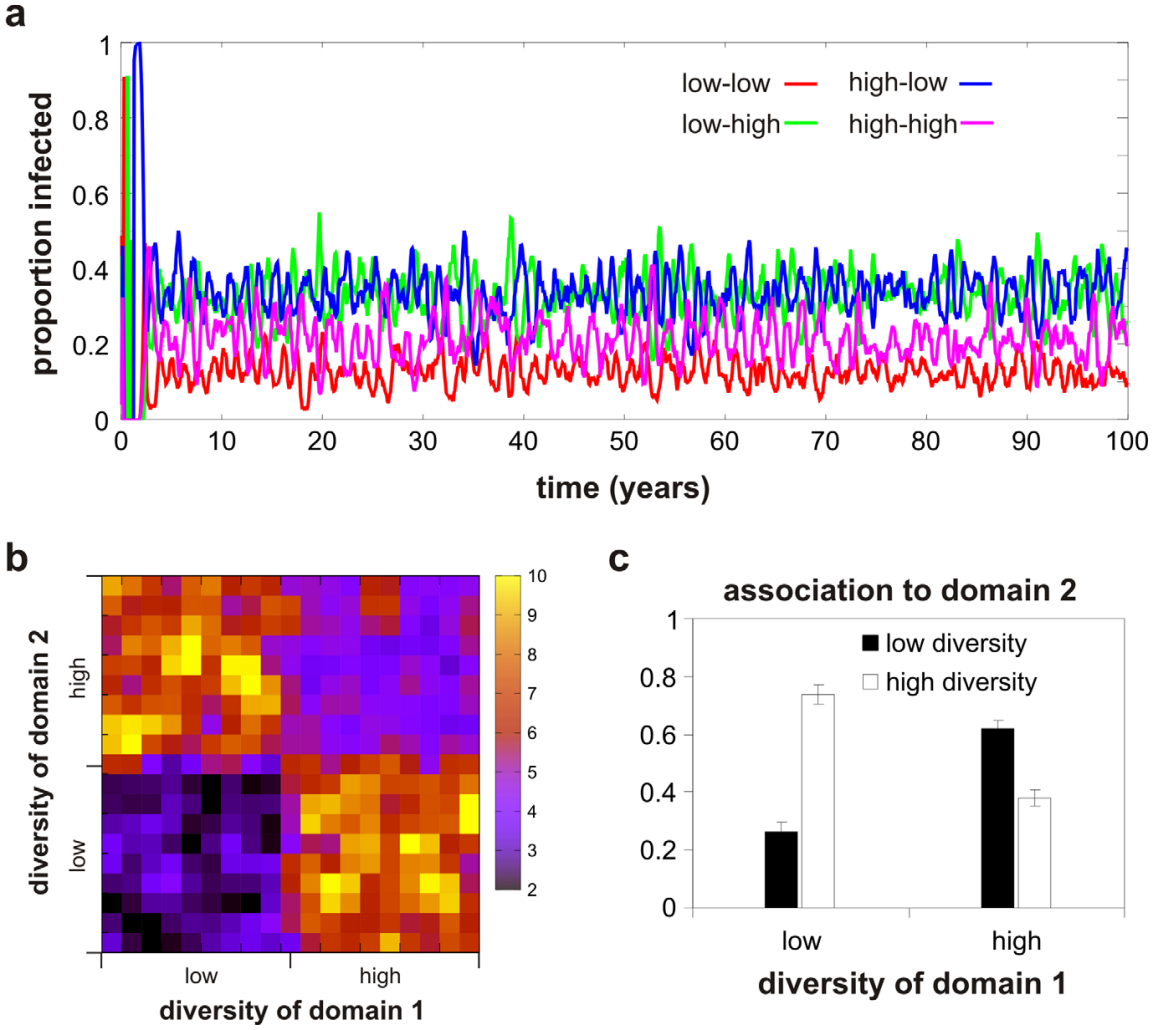
Evolution of domain associations. ***a***) Simulated timeseries of the different domain combinations grouped by the degree of diversities of their binding domains shows an evolutionary advantage and dominance of genes whose binding domains have similar levels of diversity (low-low or high-high). ***b***) Heat map of the long-term average frequency distribution of all possible domain combinations (taken from the last 30 years of the timeseries in ***a***) showing significantly higher representation of genes with similar domain diversities. ***c***) Bar graph of the long-term (30 year) average domain diversity associations from all 400 variants in the population showing a positive correlation between the levels of diversity at each binding domain.

We first analyzed the behaviour of the two-domain representation, where the fitness of a phenotype within a given infection depends on both alleles. Figure 5 shows the evolutionary trajectory and long-term frequency distribution of the various domain combinations defining phenotypes, assuming 10 alleles of both high and low diversity at each domain, given a possible 400 different phenotypes in total. For representation purposes we divided the domains into high affinity groups and low affinity groups and further classified phenotypes by their combination of domains according to these groups, resulting in four classes: phenotypes with two high affinity binding domains, phenotypes with one high and one low affinity domain (two classes in total), and phenotypes with two low affinity domains. As illustrated in the timecourse in Figure 5a, phenotypes with equal domain diversities (or affinities), i.e. either low-low or high-high diversity, start to dominate the population over those types with mixed diversities. Although a competitive advantage of phenotypes with two high affinity binding domains could be expected, it is more surprising to also observe a selective advantage of those comprising two low affinity domains, given their much reduced within-host fitness compared to phenotypes with at least one high affinity binding domain. This suggests that the within-host binding fitness advantaged gained from having one high affinity domain is immediately outweighed by the ‘antigenic’ costs this imposes onto the phenotype due to the high level of recognition in the population. In other words, phenotypes will segregate into those that either maximise binding or minimise antigenic selection pressure. The dominance of phenotypes with equal levels of diversities is also shown in the heat map in Figure 5b, which represents the long-term average prevalence levels of all phenotypes in the population (with yellow representing high prevalence and dark representing low prevalence).

The positive correlation between the degrees of diversity of the two domains, also depicted in the bar chart in Figure 5c, is in direct agreement with the observed associations in sequence diversities amongst the binding domains of *var* genes and thus corroborates the hypothesis that the evolutionary trade-off between maintaining high-affinity binding and antigenic diversification can structure *var* genes into these non-random domain combinations.

### Evolution of gene repertoire structures

**Figure 6:**
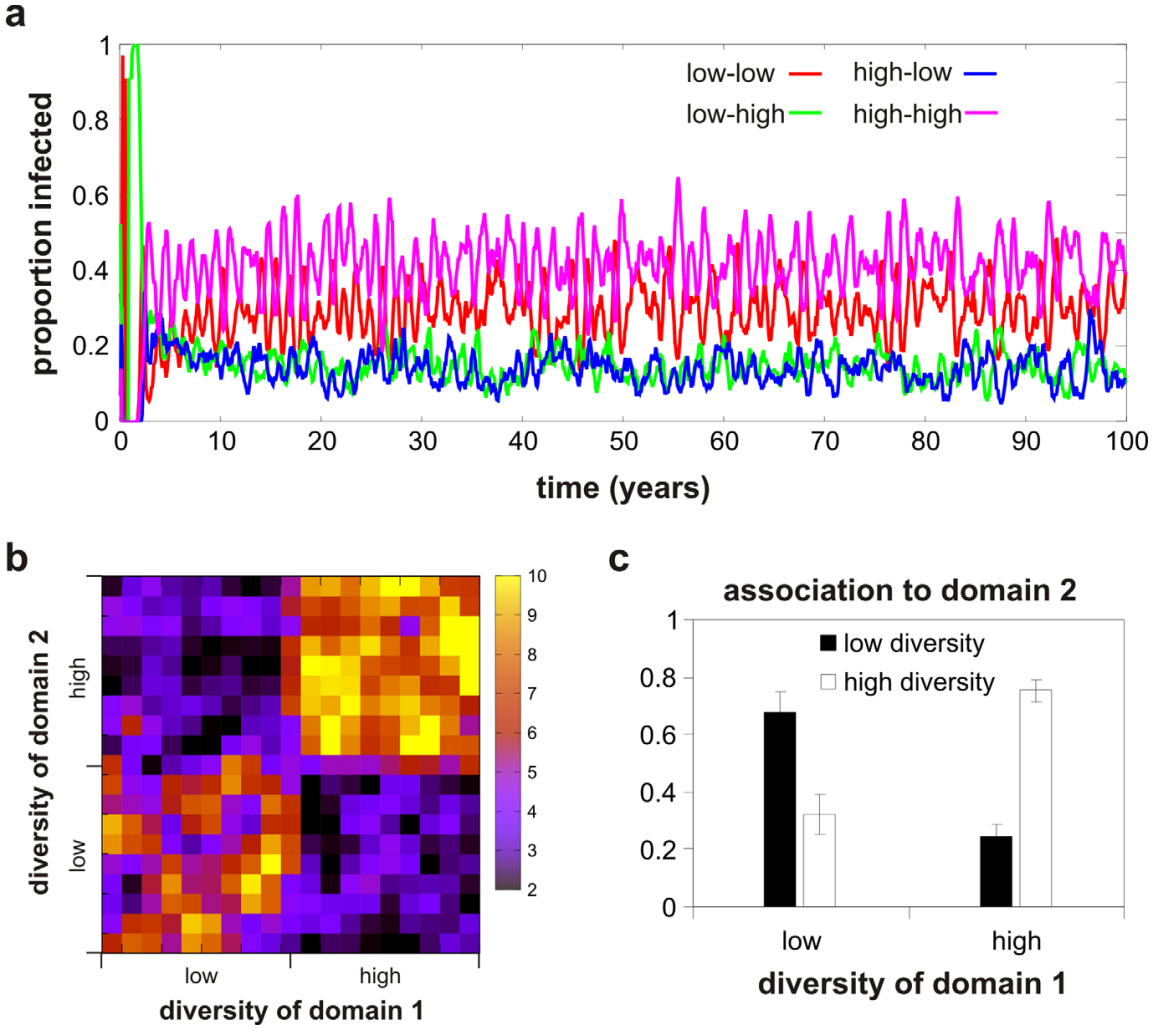
Evolution of gene associations. ***a***) Simulated timeseries of the different genotypes grouped by the degree of diversities of their defining genes shows an evolutionary advantage and dominance of genotypes whose genes have different levels of diversity (low-high or high-low). ***b***) Heat map of the long-term average frequency distribution of all possible types (taken from the last 30 years of the timeseries in ***a***) showing significantly higher representation of genotypes with different gene diversities. ***c***) Bar graph of the long-term (30 year) average gene diversity associations from all 400 variants in the population showing a negative association between the level of diversity of one gene and diversity at the second gene.

We next examined the dynamics of the two-gene repertoire model where genotypes exhibited flexible phenotype and clearance rates were determined by the “most fit” gene, dependent on binding affinity and immune history of the host. Under these circumstances we observed that the parasite population evolved towards a structure dominated by genotypes containing both high and low diversity genes, as shown in the timecourse in Figure 6a. In contrast to the previous model we now find a negative association between the levels of diversity of the genes that define a genotype, i.e. its antigenic and phenotypic repertoire, as illustrated both as a heat map of the long-term average prevalence of all genotypes in Figure 6b and as a bar graph of the different genotype classes in Figure 6c.

The finding that the most successful combination of genes is one of mixed levels of diversities is in accordance with the observation that *var* gene repertoires are generally comprised of a combination of both conserved (long) and diverse (short) genes. In other words, whereas domains of equivalent levels of diversity are evolutionarily favoured at the gene level, a mixture of conserved and diverse genes are favoured at the repertoire level. In this scenario, conserved genes are advantageous in naïve hosts through increased cytoadhesion whereas diverse genes are selected for in semi-immune hosts by means of immune evasion. This notion of a flexible phenotype allows the parasites to benefit from both strategies. Importantly, our results indicate that changing simple assumptions about the mechanisms of within-host fitness can explain the structuring at the level of both the individual gene and the gene repertoire.

## Discussion

The population structure of *P. falciparum* malaria is shaped by the interplay of different types of selection pressure on different epidemiological scales; parasites need to survive within hosts long enough to ensure transmission while also competing for susceptible hosts on a population-level. PfEMP1 plays a crucial part in both these processes because of its role in prolonging infection through antigenic variation and in the process of naturally acquired immunity. Our analyses of fully sequenced *var* gene repertoires demonstrate that the domain architectures of individual genes as well as the compositions of genomic repertoires are non-randomly structured and are maintained despite the high rates of recombination in *P. falciparum*. We hypothesised that these structures are the direct result of opposing selection pressures by which the parasite has to balance between immune evasion by means of antigenic diversification and functional integrity through high affinity iRBC binding. Here we sought to disentangle how these contrasting levels of selection could have led to the complex multi-domain gene architectures and *var* gene repertoires.

Importantly, we have provided a coherent theoretical explanation for the observation that *var* genes can generally be divided into genes that are either long and conserved or short and diverse. This contrasts predictions from traditional multi-strain models, which posit that parasites should evolve towards minimal antigenic domain structure (i.e. the fewer antigenic loci exposed the better) and/or maximal antigenic diversity, and assume that strong population-level competition is the dominant selective force structuring pathogen antigens. We hypothesised instead that immunity to *P. falciparum* is partly driven by antibodies which block cell adhesion in a domain-specific manner, reducing the parasite's within-host fitness rather than preventing infection or transmission *per se*. Not only does this assumption allow for the evolution of multi-domain antigenic variants, we also find that the balance between removal of the parasite by the inhibition of binding versus the direct killing through antibody-mediated opsonisation and phagocytosis (as in the classical multi-strain models) strongly influences the ratio of single to multi-domain phenotypes in the population.

Our results also show how similar assumptions about immunological processes acting on genes and genotypes can explain the observed structures and non-random associations among binding domains and gene repertoires. Here, synergistic interactions between domains lead to positively correlated levels of diversity across genes, whereas the flexible phenotype assumption leads to the evolution of genomic repertoires with mixed levels of diversity and a dual life-history strategy.

It has previously been suggested that discrete, independently circulating strains of *P. falciparum* may balance trade-offs between virulence and transmissibility independently [48], [49], [50], [51], with a small number of “severe” disease-causing, highly transmissible strains infecting young hosts and numerous “mild”, less transmissible strains infecting older hosts. The identification of commonly recognized isolates associated with severe disease and young host age seemed consistent with this hypothesis [53], [54], although the complex within-host dynamics of *var* genes suggested that defining malaria parasite strains at these loci may be difficult. Flexible phenotype allows genotypes to benefit from a range of life-history strategies, however, and the structuring we observed among simple two-gene repertoires reflected this ability (although we did not address virulence here): genotypes containing both a conserved and a diverse gene could optimize function in naïve hosts with one (group of) gene(s) and escape herd immunity in semi-immune hosts with another. Thus, rather than discrete strains, *var* gene repertoires contain the necessary components to optimize discrete “common” and “rare” phenotypes.

The clear differentiation of binding phenotypes among different groups of *var* genes, for example the ICAM1 and CD36 binding displayed by UpsB/C groups versus CR1 binding in the UpsA group, has led to the idea that different *var* genes are specialized for different ecological niches within the host, which may also define associated pathologies. It has recently been shown that among UpsB *var* genes, long genes are more likely to bind ICAM1 than short genes [55], suggesting not only that subsets within the Ups groups exhibit different binding specificities but also that gene length should be considered as an important sub-classifier of *var* genes in addition to promoter type or chromosomal location. This is further supported by our findings that gene length, or domain number, is strong and positively correlated with sequence conservation across genes. A prediction from our model is that the previously reported association between the expression of Ups A genes and severe disease in young children [56], [57], [58], [59] could partly be explained by the overrepresentation of long genes in this particular group.

It is likely that many other pathogen species face similar evolutionary trade-offs between within-host growth and between-host competition. For example, Frank and Bush [60] have suggested that the measles virus is optimized for high growth rates among immunologically naïve hosts, but cannot tolerate escape mutations in response to population-level immune pressure generated naturally or through vaccination. On the other hand, the influenza virus is less transmissible and affects a much broader age class but it benefits from tolerating higher mutation rates to easily evade herd immunity. Importantly, and in contrast to *P. falciparum*, among these pathogens an evolutionary strategy is associated with a particular genotype. Several bacterial pathogens exhibit a simplified version of the malaria parasite's antigenic/phenotypic variation strategy called phase variation where single antigenic determinants are expressed in an on/off fashion. For example, there is some evidence that the phase variable pilus antigen of the bacteria *Streptococcus pneumoniae*, which is thought to enhance colonization and virulence through endothelial binding, is expressed preferentially in young, immunologically naïve hosts [61]. This is consistent with the idea that optimizing growth (i.e. through expression of pilus) may conflict with the need to evade herd immunity. On the other end of the spectrum, the genome of the Africa trypanosome *Trypanosoma brucei* encodes up to 1600 VSG proteins, which form a dense coat around the extracellular pathogen and shield more conserved proteins against immune attack. *Vsg* undergo antigenic variation during infection but are thought to have no other functional role and can thus exhibit seemingly unrestricted diversity. The *var* gene repertoire of *P. falciparum* therefore encompasses a range of evolutionary strategies and constraints, representing a fascinating model system to study many aspects of host-pathogen evolution that are, on their own, likely to be common to many pathogens.

There are a number of potential caveats with our approach. First of all, there might be other functional constraints and requirements responsible for some of the genes' domain architectures, such as structural integrity or synergistic interplays of multiple domains in mediating effective binding to the same target cells. Nevertheless, given the huge variety in domain combinations and domain numbers as well as the range in sequence conservation across the *var* genes, it is difficult to imagine that these are the main or only driving force behind the observed structures and associations reported here. Secondly, in our models we only considered a very small number of binding domains or genes. Although it is easy to argue that the same selection processes should operate on higher numbers of domains and genes as well, further research will be required to show this explicitly.

Another important and necessary extension of our work would be an explicit description of within-host antigenic and phenotypic variation which should further help to distinguish between *in vivo* immune selection, intrinsic gene expression bias and (host niche dependent) growth advantages. In our framework we only considered two, antigenically unrelated genes mediating adhesion to different host receptors, and it will be crucial in the future to extend this to more genes which compete both on an antigenic as well as phenotypic level. Clearly, an understanding of the balance of immune mechanisms that modulate or protect against malaria infection (e.g. binding inhibition or opsonophagositosis) is also critical for moving forward, and our models highlight the important role that these processes play in determining the outcomes of evolutionary selection. As it stands, our results support a scenario where a small number of large *var* genes have a selective advantage in immunologically naive individuals due to their high number of relatively conserved binding domains. This advantage, however, is rapidly lost as individuals acquire a repertoire of protective antibodies which will consequently lead to the selection of genes with fewer and antigenically more diverse loci.

Generally, PfEMP1 has not been considered as a vaccine candidate due to its immense diversity. However, our growing knowledge about the involvement of individual binding domains in malaria pathology has started to open up new and exciting avenues for disease control. Efforts are already under way to evaluate and optimize *var2csa* as a target antigen for protecting pregnant women against malaria [62], . This research is based on the unusual sequence conservation of a subset of its multiple binding domains. Our results suggest that other domains to different host receptors might also exhibit conserved features due to the functional constraints of maintaining high affinity binding. Importantly, these conserved domains could potentially be shared by a much larger proportion of the global *var* gene reservoir, suggesting that PfEMP1-based vaccines might offer a realistic addition to ongoing vaccine developments, at least with regards to protection against the most severe forms of the disease.

## Methods

### Multi-domain evolution model

We assume a pathogen phenotype, defined by the expression of a particular gene, to be composed of either one or two receptor-specific binding domains. Without loss of generality, we denote domain 1 as mediating binding to receptor 1 (e.g. CR1) and domain 2 as mediating binding to receptor 2 (e.g. CD36). We further assume that each domain has a number of alleles, denoted *A_i_* and *a_i_* at binding domain 1 and 2, respectively. The dynamics and evolutionary trajectory of each phenotype *i*, carrying either a single or a two binding domains, can then be described by dividing the host population into a number of overlapping compartments: *z_i_*, the proportion that has been exposed and is now immune to phenotype *i*; 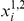, the proportion of individuals that have been exposed and are now immune to any phenotypes which share either one or two binding domains that constitute phenotype *i* (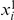 and 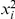, respectively); *w_i_*, the proportion of the population exposed and immune to all phenotypes that share any alleles with phenotype *i*; and finally 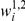, the proportion exposed and immune to phenotypes which share particular alleles at domain 1 or at domain 2 (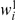 and 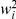, respectively). Note, under this set-up, 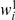 and 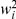 are subsets of *w_i_*, for example, and 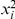 is a subset of 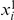.

The rate of change in these compartments can then be described by the following set of differential equations for each phenotype *i*:
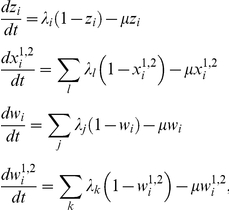
where the force of infection is given as 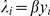, and 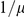 is the average host life-expectancy. The dynamics of the population currently infected and infectious with single domain phenotypes is given as
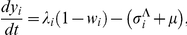
whereas for multi-domain phenotypes we use
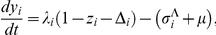
with 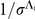 as the duration of infection of phenotype *i*, dependent on immune history Λ*_i_*. The reason for this distinction is that a single-domain phenotype can only infect hosts with no previous exposure to this particular allele (this is the proportion 1−*w_i_*), whereas multi-domain phenotypes can make use of their alternative binding domain and hence infect all hosts except those with previous exposure to both alleles that define phenotype *i*, which can be found as 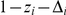, with 
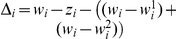
. In other words, we assume that immunity is allele specific such that antibodies to *A_i_* block adhesion of domain 1 in phenotypes carrying the *A_i_* allele and partially block adhesion of an alternative allele, *A_j_*, but do not inhibit cell-adhesion mediated by a potential second domain. Adhesion of phenotypes defined by *A_i_a_i_* will therefore not be inhibited by adhesion-blocking antibodies to just *A_i_*; on the other hand, independent exposure to alleles *A_i_* and *a_j_* will give full protection against phenotype *A_i_a_j_*.

As we do not have explicit information about the immune history of each individual host we calculate the clearance rate as a population average as follows:
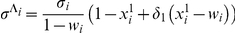
for single domain phenotypes, and
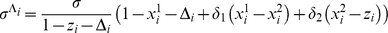
for multi-domain phenotypes, where *σ* is the base clearance rate. This means that we average the clearance rate over the proportions of the population that have not been exposed to phenotypes with any shared domains (e.g. 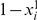 in the single domain case) and those that have been exposed to phenotypes with either one or two shared binding domains (e.g. 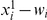, in the single domain case), which are subjected to a degrees of cross-immunity, 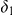 (and 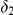, for multi-domain phenotypes, with 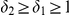).

### Individual-based model

As in the deterministic models we define a genotype or phenotype by two antigenic alleles, either representing individual domains or individual genes, which mediate binding to different host receptors. We further assume that each domain or gene has a number of alleles with different degrees of (sequence) diversity that determine both binding affinity and antigenic similarity. That is, more conserved alleles are assumed to have a higher binding specificity/affinity but also suffer from a higher degree of cross-inhibition. Antibodies against a low diversity, high-affinity allele therefore block adhesion of any of the other high-affinity alleles to a much higher degree than antibodies against a low-affinity allele, and vice versa. In addition to inhibiting cell adhesion, we also assumed cross-immunity to operate by direct killing of phenotypes or genotypes with shared or antigenically similar domains, e.g. through phagocytosis or opsonisation, albeit to a smaller extent. The within-host fitness of a pathogen is therefore dependent both on the binding affinities of its antigenic loci and immune-mediated inhibition (i.e. the immune history of the host).

We considered two different scenarios: one in which phenotypes were defined by two binding domains, and one in which genotypes were defined by two antigenic loci. These two scenarios are simply distinguished by the assumption made about how immunity mediates clearance rates. In the phenotype model, both domains act synergistically in determining the gene's fitness in terms of infection length, schematically given as 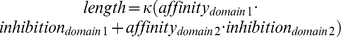, where *κ* is a positive constant. In contrast, in the genotype model, each gene is assumed to act independently and the pathogen's within-host fitness is thus defined by the fittest gene and can be given as 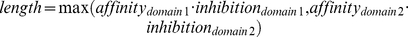. Although this at first might seem a crude assumption, for a low number of genes it is more realistic than assuming genes are being expressed in order and also more in line with a previous model of antigenic variation [46], especially as we assume no inherent immunological inhibition between the two genes.

Assuming *n* high-affinity and *m* low-affinity alleles at each of the two domains or genes, there were a total of 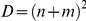 phenotypes/genotypes that are maintained in the population through recombination and occasional re-introduction to counter stochastic extinction events. We do not explicitly take into account the mosquito population but instead assume that transmission is proportional to the number of individuals infected with any of the *D* possible pathogens. We allow super-infection but for simplicity assume that infections are independent of each other, i.e. there is no within-host competition and infection length (transmissibility) is dependent only on the infecting phenotype/genotype and the host's previous immune repertoire. A list of parameter values and their definition can be found in Table S2 in the Supplementary Materials.

## Supporting Information

Figure S1 **Relationship between frequency of occurrence and sequence conservation among domain subclasses found in Ups A genes (above) and Ups B/C genes (below).** Although there is considerable overlap between groups (see Figure 2 in main text) there are domain subclasses unique to each group. The negative correlation between frequency and conservation is stronger among Ups B/C genes (*R* = −0.34, *p* = 0.0002), it is still significant among Ups A only domain subclasses (*R* = −0.28, *p* = 0.012). Note that we have removed outliers that occur in over 100 genes; these are extremely diverse and would exaggerate the correlation.
(TIF)

Figure S2 **Randomization results from 10,000 simulated data sets, where the simulated gene lengths were the same as in the data but the domains were randomized across genes.** ***a***) The distribution of correlation coefficients between gene length and average domain conservation for randomizations of the data (mean *R* = 0.01, −0.12∶0.09 5^th^∶95^th^ percentiles), with the real value (*R* = 0.49) indicated by the arrow. ***b***) The variance in population level diversity of *var* genes (using mean conservation across genes) for the randomized data (mean σ^2^ = 23, 21∶29 5^th^∶95^th^ percentiles) compared with the real value (σ^2^ = 37) indicated by the arrow. ***c***) The variance in the conservation of domains within particular genes for the randomized data (mean σ^2^ = 106.8, 103∶111 5^th^∶95^th^ percentiles), with the real value (σ^2^ = 94) indicated by the arrow. All distributions are highly significantly different from the observed value.
(TIF)

Table S1 **Pairwise correlation coefficients of the percent identity of domains at different positions within the same gene.** Starred numbers are highly significant (*P*<1E-5) following a Bonferroni correction for multiple comparisons. The purpose of this analysis is simply to show that the correlation between domains across individual genes is not simply driven by the strong relationship between particular domain combinations (for example, the head structure containing the first DBL and CIDR domains).
(DOCX)

Table S2 **Parameter values and range used in the individual-based framework.**
(DOCX)
