# Supplementary figures and images for: Evolution of the Multi-Domain Structures of Virulence Genes in the Human Malaria Parasite, Plasmodium falciparum
Source: PLoS Comput Biol. 2012 Apr 12;8(4):e1002451. doi: 10.1371/journal.pcbi.1002451 (PMC3325180; doi:10.1371/journal.pcbi.1002451)

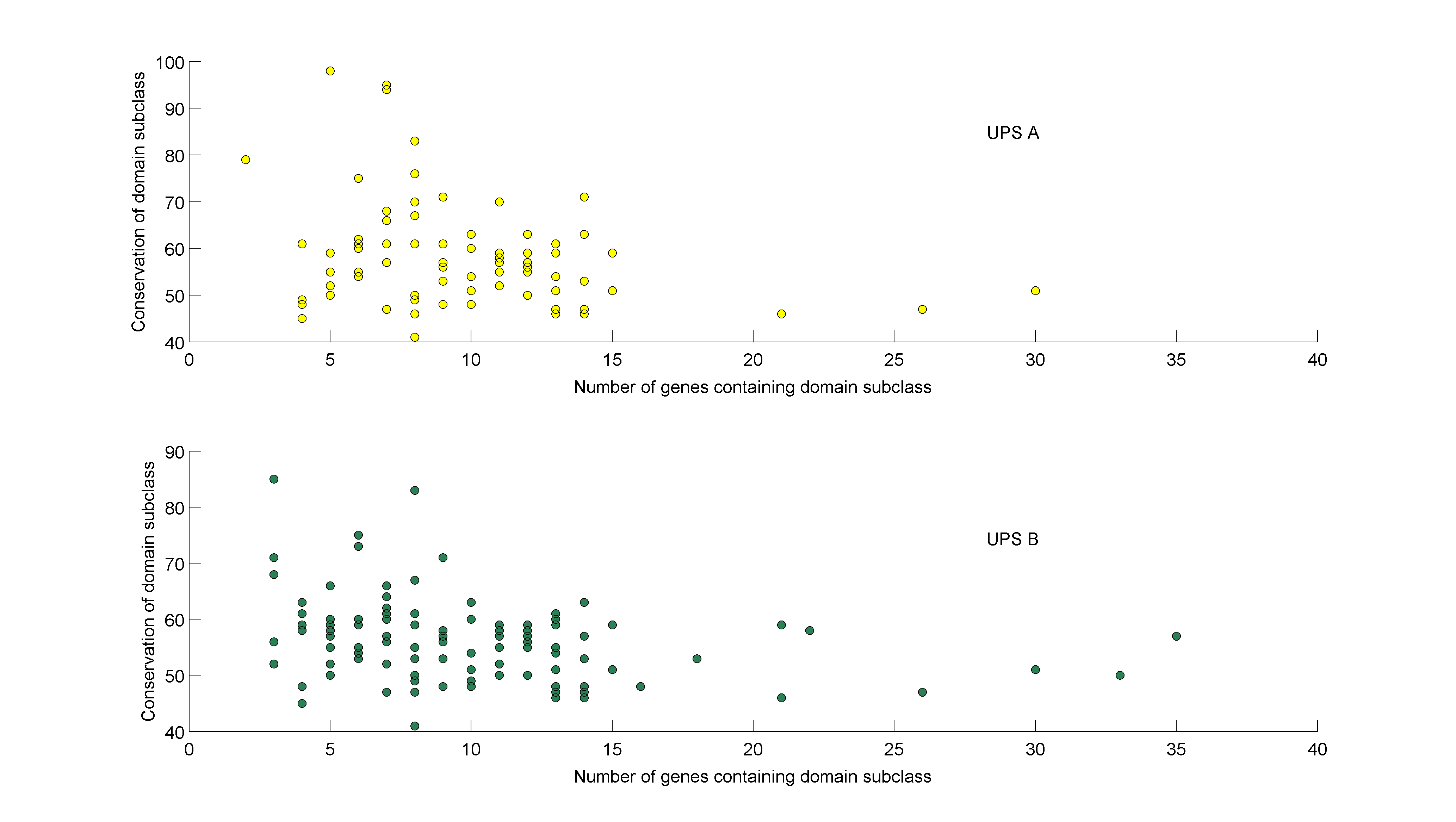

Supplement: Figure S1 — Relationship between frequency of occurrence and sequence conservation among domain subclasses found in Ups A genes (above) and Ups B/C genes (below). Although there is considerable overlap between groups (see Figure 2 in main text) there are domain subclasses unique to each group. The negative correlation between frequency and conservation is stronger among Ups B/C genes (R = −0.34, p = 0.0002), it is still significant among Ups A only domain subclasses (R = −0.28, p = 0.012). Note that we have removed outliers that occur in over 100 genes; these are extremely diverse and would exaggerate the correlation. (TIF) [file pcbi.1002451.s001.tif]

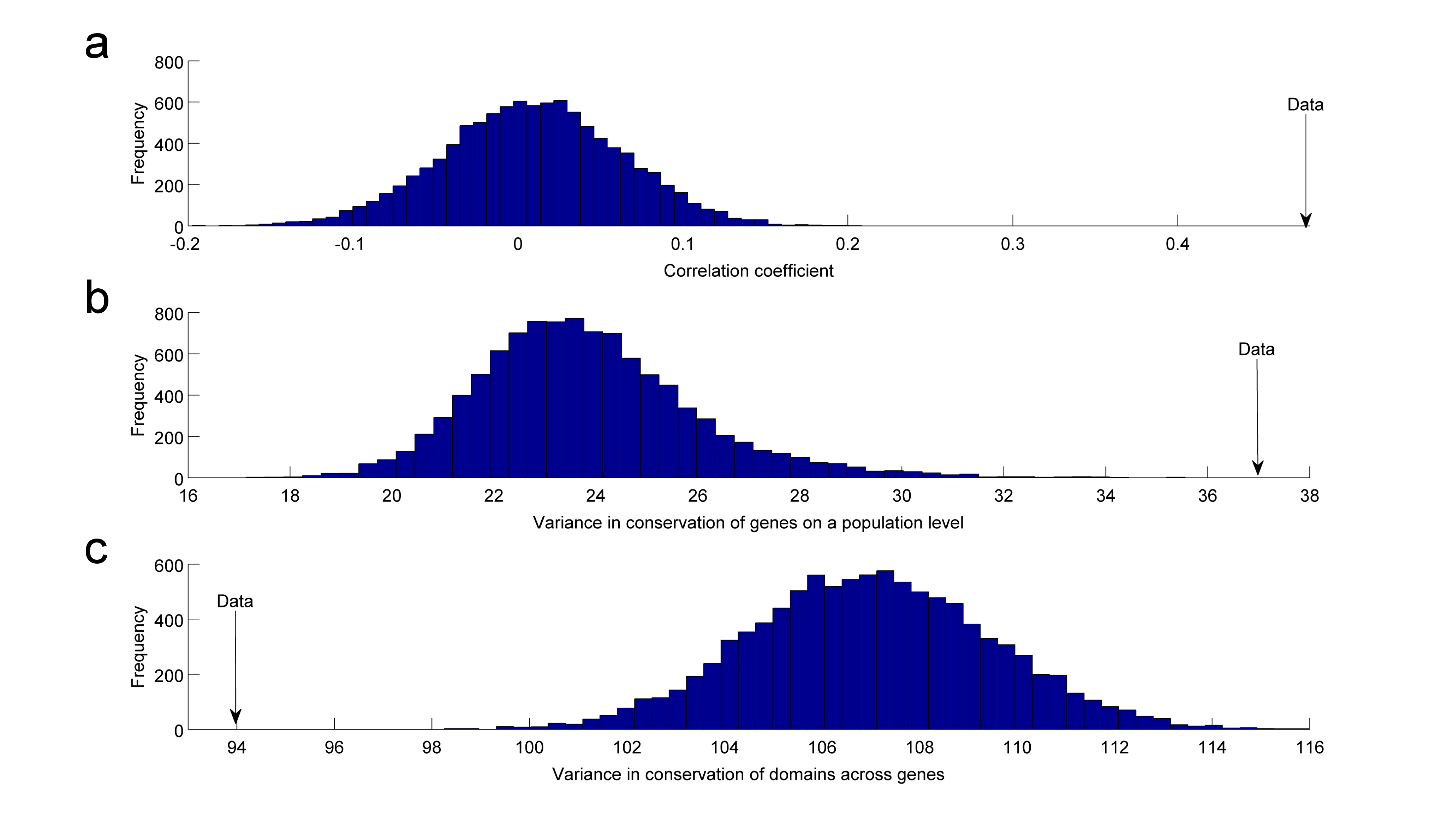

Supplement: Figure S2 — Randomization results from 10,000 simulated data sets, where the simulated gene lengths were the same as in the data but the domains were randomized across genes. a) The distribution of correlation coefficients between gene length and average domain conservation for randomizations of the data (mean R = 0.01, −0.12∶0.09 5th∶95th percentiles), with the real value (R = 0.49) indicated by the arrow. b) The variance in population level diversity of var genes (using mean conservation across genes) for the randomized data (mean σ2 = 23, 21∶29 5th∶95th percentiles) compared with the real value (σ2 = 37) indicated by the arrow. c) The variance in the conservation of domains within particular genes for the randomized data (mean σ2 = 106.8, 103∶111 5th∶95th percentiles), with the real value (σ2 = 94) indicated by the arrow. All distributions are highly significantly different from the observed value. (TIF) [file pcbi.1002451.s002.tif]
