# Supplementary material for: Evolution of the Multi-Domain Structures of Virulence Genes in the Human Malaria Parasite, Plasmodium falciparum
Source: PLoS Comput Biol. 2012 Apr 12;8(4):e1002451. doi: 10.1371/journal.pcbi.1002451 (PMC3325180; doi:10.1371/journal.pcbi.1002451)
Supplement: Table S1 — Pairwise correlation coefficients of the percent identity of domains at different positions within the same gene. Starred numbers are highly significant (P<1E-5) following a Bonferroni correction for multiple comparisons. The purpose of this analysis is simply to show that the correlation between domains across individual genes is not simply driven by the strong relationship between particular domain combinations (for example, the head structure containing the first DBL and CIDR domains). (DOCX) [file pcbi.1002451.s003.docx]

**Table S1**

| **DOMAIN** | **1** | **2** | **3** | **4** | **5** | **6** | **7** | **8** |
| --- | --- | --- | --- | --- | --- | --- | --- | --- |
| **1** | 1 | **0.386*** | **0.343*** | **0.193*** | **0.277*** | **0.297*** | *0.230* | *0.492* |
| **2** |  | 1 | **0.585*** | **0.347*** | **0.208*** | **0.392*** | *0.370* | **0.851*** |
| **3** |  |  | 1 | **0.305*** | *0.197* | **0.340*** | *0.385* | **0.668*** |
| **4** |  |  |  | 1 | **0.282*** | **0.370*** | **0.402*** | **0.721*** |
| **5** |  |  |  |  | 1 | **0.679*** | **0.541*** | **0.784*** |
| **6** |  |  |  |  |  | 1 | **0.486*** | **0.881*** |
| **7** |  |  |  |  |  |  | 1 | *0.499* |
| **8** |  |  |  |  |  |  |  | 1 |
