# Supplementary material for: Evolution of the Multi-Domain Structures of Virulence Genes in the Human Malaria Parasite, Plasmodium falciparum
Source: PLoS Comput Biol. 2012 Apr 12;8(4):e1002451. doi: 10.1371/journal.pcbi.1002451 (PMC3325180; doi:10.1371/journal.pcbi.1002451)
Supplement: Table S2 — Parameter values and range used in the individual-based framework. (DOCX) [file pcbi.1002451.s004.docx]

**Table S2.**

|  | ***parameter*** | ***value*** |
| --- | --- | --- |
| *p*_mort_ | host age-specific mortality rate | $2\cdot10^{-2}\exp\left(7\cdot10^{-2}\cdot age\right)$ |
| *β* | daily transmission probability | [0.05..1] |
| *ρ* | recombination rate | [0..0.01] |
| *κ* | proportionality factor | [0.5..1] |
| *1/σ_A,a_* | infection length mediated by high-affinity binding alleles | [50..120] days |
| *1/σ_B,b_* | infection length mediated by low-affinity binding alleles | [20..80] days |
| *N_h_* | human population size | [5000..50000] |
